# Nutritional Level and Type of Feed on the Reproductive Performance of Ewe and Its Effects on Offspring

**DOI:** 10.1111/rda.70287

**Published:** 2026-08-03

**Authors:** Fernanda Ferreira dos Santos, Maria Fernanda Soares Queiroz, Mônica Márcia da Silva, Amanda de Carvalho, Luciano Brochine, Mariluce Cardoso Oliveira, Sarita Bonagurio Gallo

**Affiliations:** ^1^ Department of Nutrition and Animal Production, Faculty of Veterinary Medicine and Animal Science University of São Paulo Pirassununga São Paulo Brazil; ^2^ Department of Animal Science and Rural Extension, Faculty of Agronomy and Animal Science Federal University of Mato Grosso Cuiabá Mato Grosso Brazil; ^3^ Department of Animal Science, Faculty of Animal Science and Food Engineering University of São Paulo Pirassununga São Paulo Brazil

**Keywords:** chromium, fetal programming, lamb, nutrition, puberty, rumen‐protected fat

## Abstract

The amount and source of nutrition are important factors that influence an ewe's reproductive ability, but the effects of diet on the reproductive performance of its offspring are unclear. This study aimed to investigate the impact of maternal nutrition (level of nutrition and source) on the reproductive performance of ewes and their female lamb offspring. Seventy‐two Dorper × Santa Inês ewes were allocated in a completely randomized block design, in five treatments: CTL (*n* = 14) with 100% of requirement, and the energy source was corn; RES (*n* = 14) with 90% of predicted requirement, and the energy source was corn; SUPP (*n* = 15) with 110% of requirement, and the energy source was corn; CR (*n* = 15) treatment SUPP plus chromium propionate; and FAT (*n* = 14) treatment SUPP plus calcium salts of palm oil. Non‐parametric data were evaluated using the Fisher exact test, and parametric data were evaluated using the Tukey test, both at a 5% significance level. There was no difference in pregnancy rate (*p* = 0.1944) or prolificacy (*p* = 0.5729) among the treatments; however, the CR diet resulted in a faster return to estrus after lambing (*p* < 0.001). The RES diet decreased lamb survival (*p* < 0.005) and reduced offspring reproductive potential. Using chromium positively affected the reproductive parameters of female offspring, whereas FAT adversely affected them. In conclusion, the source and level of dietary energy influence fetal programming in ewes. The restriction negatively affected the reproductive performance of the offspring, and supplementation with chromium propionate improved it.

## 1 Introduction

Nutrition in sheep affects several reproductive parameters, including age at puberty, ovulation rate, birth interval, embryo survival, fetal development and neonatal metabolism. The amount of energy ingested by sheep directly impacts several reproductive parameters. A negative energy balance diet results in inhibition of hypothalamic GnRH secretion, absence of LH pulses, low FSH concentrations, inhibition of folliculogenesis, low estradiol levels, increased sensitivity to negative feedback, anovulation, anestrus and delayed puberty (Scaramuzzi et al. 2006).

Corn is the main source of energy supplementation for ewes. When starch ferments in the rumen, more propionate is produced relative to acetate. This increases the amount of glucose in the blood through hepatic gluconeogenesis. However, it can decrease milk fat content and increase lactic acid production, thereby lowering rumen pH (Klevenhusen 2019). Another energy source is the calcium salts of fatty acids (protected fat), which are less affected by biohydrogenation and release more energy than starch (Silvestre et al. 2011). Chromium supplementation improves the utilization of dietary energy by facilitating insulin's action on body cells (Leiva et al. 2017; Vincent 2000, 2004).

The effects of maternal nutrition and parental impact have been studied. However, Reynolds et al. (2022) suggest that future studies should focus on the consequences of developmental programming on the life course of offspring and subsequent generations. Santos et al. (2022) reported that, despite no differences in weight gain, lambs from an undernutrition diet were heavier than those from ewes fed an overnutrition diet. Furthermore, the type of feed affects maternal filial behaviour and fetal programming.

The effects of maternal undernutrition on offspring reproductive health are likely to be observed during prolonged periods of undernutrition (Barcellos et al. 2024). The maternal diet not only affects the reproductive life of the ewe but can also alter the reproductive parameters of its offspring. Since the 1970s, researchers have recognized that metabolic changes during prenatal nutrition can affect offspring productivity after birth. Kenyon and Blair (2014) reported that studies demonstrating the effects of nutritional levels above those necessary for pregnancy maintenance on the reproductive performance of offspring are scarce.

Maternal undernutrition during the mating season and early gestation delays fetal ovarian development (Borwick et al. 2003; Rae et al. 2001) and reduces ovulation rates in adult offspring (Rae et al. 2002). Undernutrition during early and middle gestation reduces the number of ovulations in offspring (Kotsampasi et al. 2009). Excess nutrients during the early and mid‐pregnancy periods can impair the development of the ovarian follicular reserve and, consequently, reduce the reproductive potential of female foetuses (da Silva et al. 2003). This study hypothesizes that a low‐energy diet negatively affects the reproduction of ewes and their female lamb offspring. The addition of chromium propionate and rumen‐protected fat (RPF) is expected to impact the reproductive life of female lamb offspring positively. The study examined the effects of maternal nutrition on the reproductive performance of ewes and their offspring by investigating different nutrition levels (restriction, control, supplementation) and sources of energy (corn, chromium propionate and calcium salts of palm oil).

## 2 Material and Methods

The Ethics Committee for the Use of Animals at the Faculty of Veterinary Medicine and Animal Science at the University of São Paulo approved all procedures (protocol no. CEUA 2700201218). The study was conducted at the University of São Paulo, Faculty of Veterinary Medicine and Animal Science in Pirassununga, São Paulo, Brazil.

### 2.1 Ewe and Nutritional Treatments

Seventy‐two crossbreed Dorper × Santa Inês ewes, with a body weight (BW) of 59.65 ± 10 kg and 2 to 4 years old, were used. Evaluate the effects of different nutrition levels (restrictive, control and high supplementation) and different feeds (corn, chromium propionate or calcium salts of palm oil) during gestation and lactation on the reproductive performance of the ewe and its offspring.

The treatments were CTL (*n* = 14); ewe received 100% of the requirement with corn as the energy source. RES (*n* = 14), diet with 90% of the requirement with corn as the energy source. SUPP (*n* = 15), diet with 110% of the requirement with corn as the energy source. CR (*n* = 15), diet with 110% of the requirement with corn and chromium propionate as the energy sources. FAT (*n* = 14), diet with 110% of the requirement with corn and palm oil calcium salts as the energy sources (Table 1).

**TABLE 1:** Ewes' diets composition and nutritive value in early gestation, latter gestation and lactation.

	Diet treatment												
	RES	CTL			SUPP			CR			FAT		
		Early	Latter	Lact	Early	Latter	Lact	Early	Latter	Lact	Early	Latter	Lact
Ingredient (% DM)													
Hay	99.00												
Corn silage		85.00	70.00	60.00	68.00	50.00	41.00	68.00	50.00	41.00	72.00	58.00	47.00
Ground corn		9.00	19.00	26.00	24.00	37.00	44.00	24.00	37.00	44.00	18.00	26.00	35.00
Soybean meal		5.00	10.00	13.00	6.00	11.00	13.00	6.00	11.00	13.00	7.00	12.00	14.00
Rumen protected fat											2.00	3.00	3.00
Chromium								0.01	0.01	0.01			
Mineral mix	1.00	1.00	1.00	1.00	1.00	1.00	1.00	1.00	1.00	1.00	1.00	1.00	1.00
LimeSUPPone					1.00	1.00	1.00	1.00	1.00	1.00			
Chemical composition (%)													
Dry matter (%)	89.00	29.10	32.83	36.08	33.50	39.89	44.47	33.50	39.89	44.47	32.44	36.96	41.53
Crude protein (% DM)	7.23	8.80	10.55	11.84	9.55	11.14	12.19	9.55	11.14	12.19	9.46	11.10	12.00
Ether extract (% DM)	2.28	1.58	1.89	2.10	2.01	2.42	2.65	2.01	2.42	2.65	3.57	4.61	4.65
Metabolizable energy (Mcal)	1.88	2.06	2.24	2.37	2.26	2.46	2.60	2.26	2.46	2.60	2.27	2.47	2.60
Acid detergent fibre (% DM)	40.58	38.18	32.63	28.74	31.46	24.53	20.83	31.46	24.53	20.83	32.94	27.58	23.23
Neutral detergent fibre (% DM)	71.57	61.08	52.62	46.68	50.99	40.48	34.88	50.99	40.48	34.88	53.12	44.89	38.36
Mineral matter (% DM)	7.84	7.29	6.72	6.26	7.04	6.72	5.96	7.10	6.90	6.00	6.89	6.47	5.84
Calcium (% DM)	0.65	0.53	0.48	0.42	0.67	0.75	0.60	0.67	0.75	0.60	0.71	0.77	0.67
Phosforus (% DM)	0.31	0.27	0.32	0.32	0.29	0.32	0.33	0.29	0.32	0.33	0.28	0.31	0.32

*Note:* RES: 90% of requirement and the energy source was corn; CTL: 100% of requirement and the energy source was corn; SUPP: 110% of requirement and the energy source was corn, CR: SUPP more chromium propionate and FAT: SUPP more palm oil rumen protected fat.

The ewes were fed in a feedlot in collective pens, with 3 or 4 ewes per pen and four pens (replications) per treatment. The feed was offered twice daily (at 8 and 16 h) throughout gestation (150 days) and lactation (70 days). Dry matter consumption (DMI) was calculated per pen using the equation described by Gallo and Tedeschi (2021).

The energy requirement was calculated using this equation by NRC (2007):

(1) $$\text{MEpreg}\;\left(\text{Mcal}/\mathrm{day}\right)=\left(36.9644\;x\;e^{(-11.465\;xe^{\left(-0.00643\;\mathrm{xT}\right)-0.00643\;x\;T)}}x\;\left(\frac{\mathrm{LBW}}{4}\right)/0.13\right]$$

In which ME is metabolizable energy during pregnancy, *T* is the gestation length in days and LBW is the lamb's birth weight in kg.

For lactating ewes, the determination of the metabolizable energy according to NRC (2007) was represented in Equations (2) and (3):

(2) $${\mathrm{NE}}_{\mathrm{LR}}=\mathrm{MY}\;x\;\left(0.25173+0.08964\;x\;\mathrm{MkF}+0.03785\;x\;\text{MkTP}/0.93\right)$$

(3) $${\mathrm{ME}}_{\mathrm{LR}}={\mathrm{NE}}_{\mathrm{LR}}/0.644$$

In which: MkF is the milk fat content, g/100 g; MkTP is the milk total protein, g/100 g; MY is the total milk production, kg/day. Protein and fat levels of 5% and 7%, respectively, in milk were considered.

The lambing date was estimated from the mating date, and the pregnancy was evaluated by ultrasound. The requirement for crude protein, calcium and phosphorus was as recommended by the NRC (2007).

#### 2.1.1 Ewes Management

Ewes were treated with a controlled intra‐vaginal drug release device (Eazi‐breed CIDR Sheep and Goat Device, 0.33 mg progesterone in inert silicone elastomer—ZOETIS Veterinary Products Industries Ltda) for 9 days. On the 7th day, 1.25 mL (250 UI) pregnant mare serum gonadotropin (PMSG; Novormon ZOETIS Veterinary Products Industries Ltda) and 2.0 mL prostaglandin (Cloprostenol sodium 25 mg; Sincrocio Ouro Fino Animal Health) were injected for better estrus synchronization (Uriol et al. 2019). After 24 h CIDR removal, ewes were put together with a male for controlled breeding: 1 male with 5–6 females, and mating was observed. Rectal ultrasound on Day 30 after breeding was performed to determine pregnancy rate (number of ewes pregnant per ewe in mating), and prolificacy was calculated on the number of lambs born per ewe.

### 2.2 Offspring Management

During the birth season, all animals were inspected during the day (6 to 19 h) to record ewe and lamb identity, litter size, birth weight, sex and to dip the navel of each lamb in a 10% iodine solution. The offspring had access to creep feeding, and the concentrate contained 22% crude protein and 3.2 Mcal of ME. The feed with corn silage and concentrate was offered ad libitum.

Lamb survival rate was defined and analyzed in proportion of birth; proportion of lambs alive on Day 7 after birth; proportion of lambs alive on Day 7 of total born; proportion of lambs alive on weaning of born alive, and proportion of lambs alive on weaning of total born.

After weaning (70 days), female lambs (*n* = 46) were kept together, with the distribution across treatments as follows: CTL to 8, RES to 7, SUPP to 11, CR to 11 and FAT to 9. All female lambs were fed with corn silage, corn grain and soybean meal to meet their growth requirements (NRC 2007). The total diet contained 14% crude protein and 2.9 Mcal of ME. Female lamb survival was defined as the proportion of female lambs alive at the breeding season out of those alive at weaning.

#### 2.2.1 Management of Reproduction in Female Lambs

At 7 months of age, females with a minimum BW of 40 kg, corresponding to 60%–70% of their adult weight, were selected for the breeding season and considered replacement animals, ensuring they were at puberty and fit for reproduction. The number of animals that met these criteria was: CTL: 7; RES: 6; SUPP: 10; CR: 10; and FAT: 8, totaling 41 sheep.

Throughout this experimental period, the animals' diet was monitored daily, with a total feed intake of 14% crude protein and 2.9 Mcal of metabolizable energy. The feed provided consisted of Cost‐cross hay, a concentrate made from finely ground corn and soybean meal, and supplements of macro‐ and microminerals.

For the breeding season, two White Dorper rams were introduced into the paddock of young ewes for 34 days. Each ram was fitted with a crayon harness (different colours), and on Day 17, the crayon colour was changed to allow detection of females that did not conceive, expressed a second estrus and re‐mated. In that way, it was possible to determine the mating day.

Transrectal ultrasound evaluations were performed on Day 28 after mating to diagnose pregnancy (Moraes et al. 2009). On Days 40 and 60 of pregnancy, an ultrasound was carried out to determine embryonic and fetal death, respectively. Late embryonic death occurs between 30 and 40 days of gestation, and after that, it is considered fetal death (Yotov 2012). In each treatment, pregnancy rate and prolificacy were calculated.

At birth, the lamb's identity, litter size, birth weight, sex and mortality were recorded, and the navel of each lamb was dipped in a 10% iodine solution.

### 2.3 Blood Samples and Hormone Levels of Ewes and Offspring

Blood samples were collected from the jugular vein of all ewes during the estrus synchronization (*n* = 72) and for estrus return (*n* = 71), and for puberty of all female lamb offspring (*n* = 37) into 9 mL vacutainers with no anticoagulant. Samples were centrifuged at 1500xg for 15 min and serum separated and stored at −20°C until analysis for estradiol (E2) and progesterone (P4).

Using synchronization protocols with P4 and equine chorionic gonadotropin (eCG), the onset of estrus behaviour is 34 h after removing the CIDR (Uriol et al. 2019). Therefore, for E2 analyses during estrus synchronization, blood was collected at −12, 0, 12, 24 and 36 h, with 0 h marking ovulation. For progesterone analysis during this time, blood was collected on Days 1, 4 and 7 after ovulation to assess the formation and function of the corpus luteum. To verify the return of estrus (when progesterone levels exceed 1 ng/mL), blood samples were collected twice weekly from Days 30 to 60 after lambing.

The concentration of serum progesterone in female offspring was evaluated weekly from 20 weeks of age to determine when they reached puberty (progesterone > 1.0 ng/mL). To measure serum progesterone, the radioimmunoassay technique (RIE) in solid phase was used, using a commercial diagnostic kit from Siemens (COAT—A—COUNT, Diagnostic Products Corporation, Los Angeles, CA, USA) developed for quantitative evaluation of progesterone in human serum, and previously validated for use in sheep serum. The quantification of serum oestrogen was performed using the RIE technique, with a diagnostic set of double‐stranded 3rd generation estradiol from DSL‐39,100 (Diagnostic System Laboratories, Webster, Texas, USA), due to its ultra‐sensitivity.

### 2.4 Statistical Analyses

The experimental design was a completely randomized block design, with animals blocked according to parturition order (nulliparous and pluriparous). The following response variables were analyzed under this model: hormonal concentrations (e.g., progesterone and estradiol) and prolificacy. The effect of the interaction between the experimental treatment and the type of pregnancy was evaluated. Data were analyzed using SAS version 9.1.2 for Windows (SAS Institute 2012).

Prior to analysis, assumptions of normality and homogeneity of variances were evaluated using the Shapiro–Wilk and Levene tests, respectively. When these assumptions were met, data were analyzed using ANOVA (PROC GLM), including treatment as a fixed effect and block (parturition order) as a random effect. When significant effects were detected (*p* < 0.05), means were compared using Tukey's test.

Reproductive indices (pregnancy rate, estrus return, survival, puberty attainment, suitability for reproduction and abortion) were analyzed as categorical variables using PROC FREQ and compared by Fisher's exact test at a 5% significance level. Puberty was analyzed using this approach because it was defined as a binary outcome (attained vs. not attained), rather than a continuous variable.

## 3 Results

### 3.1 Ewes

There was no difference in the percentage of pregnant sheep (*p =* 0.1944) or prolificacy (*p =* 0.5729) between treatments. However, the percentage of sheep that returned to estrus between 30 and 60 days after parturition differed among the diet groups (*p <* 0.001). Fifty‐three percent of the ewes on CR were able to return to estrus, compared to 7.7% and 14.3% of the ewes on RES and CTL, respectively (Table 2).

**TABLE 2:** Ewe fed different diets and energy levels, and their effects on reproductive parameters and the survival rate of their offspring.

	Ewe nutrition treatment					*p*
	RES	CTL	SUPP	CR	FAT	
Pregnancy rate, %	100	100	100	100	92.85	NS
Prolificacy rate, %	1.43 ± 0.06	1.36 ± 0.06	1.53 ± 0.06	1.53 ± 0.06	1.23 ± 0.06	*
Return to estrus in 70 to 90 days post partum, %	7.69^b^	14.28^b^	35.71^ab^	53.33^a^	38.46^ab^	*
Birth, %	90^b^	100^a^	100^a^	100^a^	100^a^	*
Survival rate, %						
Day 7	88.8^b^	100^a^	100^a^	100^a^	100^a^	*
Day 70, weaning	88.8^b^	100^a^	100^a^	100^a^	100^a^	*

*Note:* RES: 90% of requirement and the energy source was corn; CTL: 100% of requirement and the energy source was corn; SUPP: 110% of requirement and the energy source was corn, CR: SUPP more chromium propionate and FAT: SUPP more palm oil rumen protected fat. Prolificacy: lambs born/ewe; Estrus: estrus return after lambing. *Letters on the column differ from the averages by the Fisher test at 5% probability for pregnancy, estrus, birth, survival rate and by Tukey test at 5% probability for prolificacy.

**FIGURE 1:**
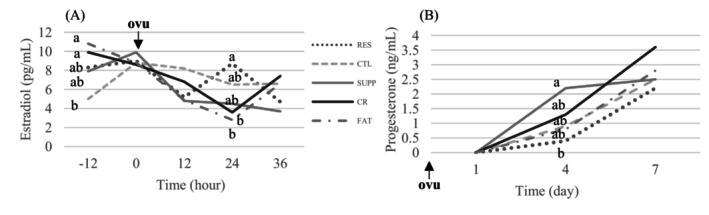
Hormonal concentration of estradiol (A) and progesterone (B) after estrus synchronization from ewes with different levels and source of energy in the diet. RES (*n* = 14): 90% of requirement and the energy source was corn; CTL (*n* = 14): 100% of requirement and the energy source was corn; SUPP (*n* = 15): 110% of requirement and the energy source was corn, CR (*n* = 15): SUPP more chromium propionate and FAT (*n* = 14): SUPP more palm oil rumen protected fat. Letters on the line differ from the averages by the Tukey test at 5% probability. OVU, ovulation time.

After the estrus synchronization, the serum E2 concentration differed among treatments at −12 h (*p =* 0.0234), with the highest concentration in ewes on FAT and the lowest in CTL. The difference remained significant at 24 h (*p =* 0.0273) after ovulation, with the highest concentration in RES ewes and the lowest in FAT (Figure 1A). Progesterone differs only on Day 4 (*p =* 0.0534), with the SUPP treatment having the highest concentration and RES having the lowest concentration (Figure 1B). There are no differences in E2 at ovulation (*p =* 0.8711), 12 h (*p =* 0.1663) and 36 h (*p =* 0.2005) after ovulation, nor in P4 at Days 1 (*p =* 0.5686) or 7 (*p =* 0.3386).

Lambs born from RES ewes had lower survival rates for birth (*p* = 0.0027, Table 3), alive d7 (*p* = 0.0038), and alive at weaning (*p* = 0.0038). Litter size (single or multiple) alone did not influence lamb survival, but the interaction between the RES group and multiple litters showed higher mortality on d0 (20%), d7 (13%) and at weaning (13%) among all lambs born (*p <* 0.001).

**TABLE 3:** The effect of gestation type on the nutrition of ewes fed different diets and energy levels on the survival rate of their offspring.

Survival rate	Ewe nutrition treatment					*p*
	RES	CTL	SUPP	CR	FAT	
At birth						
Simple	100	100	100	100	100	NS
Multiples	80^b^	100^a^	100^a^	100^a^	100^a^	*
Day 7						
Simple	90^a^	100^a^	100^a^	100^a^	100^a^	*
Multiples	87^b^	100^a^	100^a^	100^a^	100^a^	*
Day 70, weaning						
Simple	90^a^	100^a^	100^a^	100^a^	100^a^	*
Multiples	87^b^	100^a^	100^a^	100^a^	100^a^	*

*Note:* RES: 90% of requirement and the energy source was corn; CTL: 100% of requirement and the energy source was corn; SUPP: 110% of requirement and the energy source was corn, CR: SUPP more chromium propionate and FAT: SUPP more palm oil rumen protected fat. Prolificacy: lambs born/ewe; Estrus: estrus return after lambing. *Letters on the column differ from the averages by the Fisher test at 5% probability for survival rate.

### 3.2 Female Offspring

**FIGURE 2:**
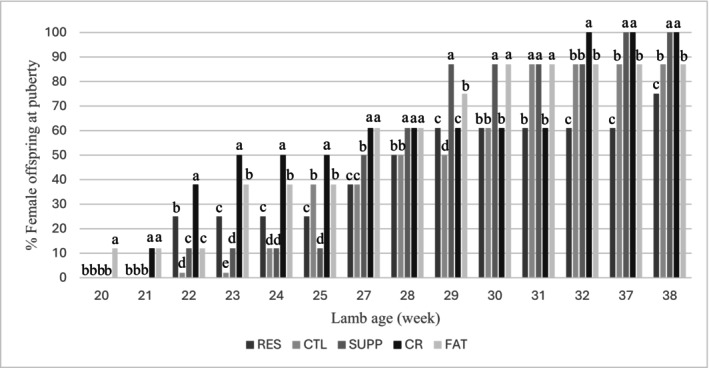
Sum of the percentage of female offspring on puberty between Weeks 20 and 38 of life, born from ewes with different levels and source of energy in the diet. RES (*n* = 14): 90% of requirement and the energy source was corn; CTL (*n* = 14): 100% of requirement and the energy source was corn; SUPP (*n* = 15): 110% of requirement and the energy source was corn; CR (*n* = 15): SUPP more chromium propionate and FAT (*n* = 14): SUPP more palm oil rumen‐protected fat. Letters on the column differ from the averages by the Fisher test at 5% probability.

The concentration of serum progesterone in female offspring was evaluated weekly from 20 weeks of age to determine when they reached puberty (progesterone > 1.0 ng/mL). Figure 2 shows the sum of the percentage of females who reached puberty between Weeks 20 and 38. The diets of the dams influenced the onset of puberty in their female lambs (*p* < 0.005).

Between Weeks 22 and 25, a higher percentage of female lambs reached puberty in the CR groups than in any other group (Figure 2), with 37.5% of CR female lambs in puberty by Week 22, against 25% RES, 12.5% FAT and SUPP and 0% CTL (*p* = 0.0054). From Weeks 23 to 25, 50% of the CR group reached puberty, compared to 37.5% FAT, 25% RES group, 12.5% SUPP group, and 0%, 12.5% and 37.5% of the CTL in Weeks 23, 24 and 25, respectively (*p* = 0.001).

In Weeks 32 (*p* = 0.0026), 37 (*p* = 0.0013) and 38 (*p* = 0.0033), the RES group had the lowest percentage of female offspring that were in puberty (62.5%, 62.5% and 75%, respectively). All CR lambs achieved puberty by Week 32, while all SUPP young ewes achieved puberty by Week 37. By the final week of measurements, 87.5% of the young ewes from the CTL and FAT groups were sexually mature, while only 70% of the young ewes from the RES group were, which was significantly different (*p* = 0.0003). Therefore, the CR and SUPP treatments had a positive effect on anticipating puberty, especially CR. The CTL, FAT and RES treatments had negative effects.

The survival rate was calculated for all the lambs alive at weaning on Day 240 (Table 4), and the difference was significant (*p* = 0.0095). SUPP and FAT had a 100% survival rate, while RES and CR had a 75% survival rate. During this period, the animals were affected by worms (*Haemonchus contortus* and *Moniezia* sp.) which can result in mortality. On day 240, females weighing over 40 kg were selected for reproduction (Table 4). All young ewes from mothers on the CTL, SUPP and CR diets were ready for reproduction, while 75% of the lambs from FAT and only 66.7% from the RES diet were ready for reproduction (*p =* 0.0080). There were significant differences in pregnancy rates (*p* = 0.0214) and abortion rates (*p* = 0.005) but not in prolificacy (*p* = 0.452) (Table 4).

**TABLE 4:** Reproductive traits of female lambs born from ewes fed with different levels and source of energy in the diet.

Results—female offspring	Ewe nutrition treatment					*p*
	RES	CTL	SUPP	CR	FAT	
Survival % Day 240/total alive on weaning	75^b^	87.5^ab^	100^a^	75^b^	100^a^	*
Available for reproduction, %^†^	66.7^c^	100^a^	100^a^	100^a^	75^b^	*
Pregnancy rate, %	75^b^	100^a^	75^b^	83^ab^	100^a^	*
Abortion, %	33.3^b^	0^a^	0^a^	0^a^	0^a^	*

*Note:* RES (*n* = 14): 90% of requirement and the energy source was corn; CTL (*n* = 14): 100% of requirement and the energy source was corn; SUPP (*n* = 15): 110% of requirement and the energy source was corn, CR (*n* = 15): SUPP more chromium propionate and FAT (*n* = 14): SUPP more palm oil rumen protected fat. *:Letters on the column differ from the averages by the Fisher test at 5% probability.

^†^ Female lambs at 8 months and more than 40 kg.

The pregnancy rates were 100% for CTL and FAT, which were different from RES and SUPP at 75%, but not different from CR (83%). There was one abortion in the RES treatment group. The abortion rate was 33% for female offspring from the RES diet and 0% for the CTL, SUPP, CR and FAT diets.

## 4 Discussion

This study indicates that diets with more available energy can positively affect the reproductive parameters of ewes and their female offspring. While there were no significant differences in pregnancy rate or prolificacy, the diet can accelerate the return to estrus after lambing, particularly when supplemented with chromium. Compared with a control diet, supplementation with fat did not affect the number of days postpartum to the first progesterone rise or ovulation (Oqla et al. 2004; Titi et al. 2008).

This study revealed differences in the treatments for return to estrus after lambing. From 7.69% to 53.33% of ewes ovulated within 60 days after lambing, for RES and CR, respectively. For non‐seasonal sheep breeds, such as the Santa Inês, to produce more lambs throughout their lifetime, it is necessary to reduce the lambing interval to obtain three births in 2 years. The duration of the anestrous postpartum is an important economic factor because a shorter period between birth and the onset of ovarian cyclicity enables conception after lambing. Nutrition plays an important role in follicular development and consequently influences the period from lambing until the first estrus and ovulation (Ascari et al. 2016). During the early postpartum period, adequate nutritional support is necessary for animals to resume ovarian activity. Nutritional supplementation with high‐energy diets stimulates folliculogenesis, which decreases negative feedback and induces compensatory increases in folliculogenesis (Ascari et al. 2016; Crowe 2008).

Delayed resumption of ovulation is invariably due to a lack of LH pulse frequency which can result from metabolic stressors (Crowe 2008; Crowe et al. 2014). In this study, diets with fewer energy were not able to resume ovarian activity within the expected 60 days. The effects of low‐energy diets on reproduction primarily occur at the hypothalamic–pituitary level of reproductive control. These effects are characterized by suppressed plasma insulin‐like growth factor 1 (IGF‐1) and elevated plasma growth hormone (GH), which are associated with anovulation and anestrus in females (Scaramuzzi et al. 2006; Wade and Jones 2004).

Among the interactions between nutrition and reproduction in mammals, increased dry matter intake (DMI) in sheep was found to decrease circulating progesterone (P4) levels and reduce embryo survival. High energy intake decreases circulating P4 concentrations, likely due to an increased liver blood flow (Jing et al. 2017; Mahmoud et al. 2012; Vonnahme et al. 2013). Vonnahme et al. (2013) found that ewes with higher feed intake had lower circulating cortisol levels, which argues against a generalized effect on hepatic steroid metabolism. A diet with 30% more energy had no effect on E2 and P4 concentrations (Mahmoud et al. 2012), though supplementation at a higher energy level increased P4 and E2 concentrations (Jing et al. 2017). However, our study did not show a reduction in plasma E2 or P4 with SUPP, CR or FAT treatments. This may be because there was no difference in DMI, only in the type of feed available in each diet (dos Santos et al. 2022).

Ewes that received the lowest‐energy diet (RES) had a lower percentage of surviving lambs until weaning, which had a greater impact on multiple births. The ewes' diet also influenced lamb survival from weaning to puberty similarly, except for the CR treatment, which had a problem with worms. Animals in the CR group did not respond effectively to the use of anthelmintics in the control of internal parasites. Successful lamb rearing for slaughter and replacement of breeding stock is a key to profitable sheep enterprises (Perry and Copping 2019), and according to these authors, the greatest losses in ruminant production systems occur during the neonatal period. Maternal undernutrition restricts intrauterine growth and is a leading cause of fetal mortality and morbidity (Gao et al. 2014). Studies show that diets with increased energy intake near the day of insemination do not influence lamb survival, probably because the supplementation period is short. However, multiple births result in fewer surviving lambs (Kleemann et al. 2015), and lamb mortality decreases when ewes are supplemented with carbohydrates or fat (Gulliver et al. 2012). This is likely due to a higher total energy intake. Nutrient restriction may affect neonatal survival by influencing dystocia via placental dysfunction, thermoregulation after birth, modification of the developing immune system and modification of maternal and neonatal behaviour (Perry and Copping 2019).

The term ‘programming’ describes the process by which a stimulus (good or bad) during fetal development has permanent effects on the physiology, structure and metabolism of different systems (Mossa et al. 2015; Perry and Copping 2019; Reynolds et al. 2019). However, some studies have investigated the impact of the maternal environment on the reproductive potential of offspring. (Mossa et al. 2015) suggested that in utero undernutrition of female ovine foetuses during the first and second third of gestation causes delayed in ovarian development and reduced ovulation rates in adulthood. Similarly, overnutrition may impair the development of the ovarian follicular reserve and consequently, the reproductive potential of female foetuses.

In this study, low‐energy diets (RES) negatively affected the reproduction of female offspring. These diets produced fewer replacement ewes, lower pregnancy rates and higher abortion rates, and also delayed puberty. These results show that undernutrition can decrease the reproductive potential of offspring.

The use of protected fat in the rumen improves maternal condition and is correlated with greater liver development in offspring, increased fetal liver global DNA methylation; FS increased ALOX5AP mRNA and FS‐MS decreased TNF‐α, PPARδ and PPARγ mRNA; MS decreased DNMT1 mRNA in fetal liver (Rosa Velazquez et al. 2020). Protected fat in the rumen during late gestation altered maternal lipid metabolism gene expression and increased myogenic mRNA in offspring. Therefore, evidence shows alterations in mRNA (Miranda et al. 2023). A specific upregulation of lipid metabolism transcripts in maternal adipose tissue and liver, associated with supplementation with calcium salts from soybean oil, has been reported. Molecular evidence points to an alteration in myogenic gene expression in lambs born to ewes supplemented with fat, consistent with altered fetal muscle development. The combined results suggest a plausible pathway in which protected fat in the maternal rumen modifies maternal lipid metabolism gene expression and nutrient delivery, which is associated with altered myogenic transcription in the offspring, but direct mechanistic evidence beyond mRNA is limited.

Of particular interest is the evidence suggesting that RPF supplementation can negatively affect fetal programming. Protected palm oil delayed female puberty, and by 8 months of age, < 90% of female offspring had reached puberty, and only 75% of them were suitable for reproduction based on their weight. Increasing the precursor of fatty acids has been well documented to lead to increased steroid synthesis, especially of eicosapentaenoic acid (C20:5; EPA) and docosahexaenoic acid (C22:6; DHA), two crucial components of steroidogenesis. Feeding supplemental fatty acids provides a higher level of energy and positively impacts reproductive performance by controlling ovarian function and follicular development (Mirzaei‐Alamouti et al. 2018). However, studies comparing palm oil (protected or not) with other oils or no supplementation have shown that palm oil lacks the benefits of supplementing with protected fat (Asgari Safdar et al. 2017; Abdel‐Hakim et al. 2016; Mirzaei‐Alamouti et al. 2018).

Supplementation with chromium positively affects ewe reproduction and the reproduction of their female offspring. In this study, more than half of the ewes that received chromium propionate were able for mating 60 days after lambing, and 100% of their female offspring achieved puberty before Week 32. This improved the cyclicality of both.

In lactating cows, chromium supplementation reduced the number of days to first estrus (63 vs. 75 days) and to first artificial insemination (71 vs. 90 days), and reduced the number of cows that did not conceive (Yang et al. 1996). Beef cows that received supplemental chromium tended to have higher pregnancy rates, suggesting that chromium may improve fertility (Stahlhut et al. 2006). Chromium supplementation increased the percentage of pregnant periparturient cows within the first 28 days of the breeding period when given under heat stress conditions (Soltan 2010). The number of days to first ovulation after calving tended to be lower for cows fed a chromium‐supplemented diet (Kafilzadeh et al. 2012). The use of chromium in fetal programming in sheep resulted in heavier lambs at slaughter (Brochine et al. 2023; Gallo et al. 2025) and up to puberty age (dos Santos et al. 2022).

## 5 Conclusion

This study shows that it is possible to program the fetus's reproductive performance during its life by manipulating the mother diet. Providing diets with energy below the recommended is not advisable due to its negative effects on the reproduction of ewes, the survival of lambs and the reproduction of their female offspring. Using RPF from palm oil negatively affects fetal programming by delaying puberty and decreasing the number of replacement ewes, suggesting that other oil sources should be used. In summary, our results contribute to the emerging evidence that chromium propionate supplementation enhances reproductive performance and developmental benefits in ewes, potentially reducing the interval to estrus after lambing, as well as affecting fetal programming by advancing female offspring puberty.

## Author Contributions

**Fernanda Ferreira dos Santos, Luciano Brochine, Mônica Marcia da Silva, Mariluce Cardoso Oliveira:** conception and design of the work, analysis, interpretation of data for the work; drafting the work and reviewing it critically for important intellectual content. **Maria Fernanda Soares Queiroz and Amanda de Carvaho:** drafting the work or reviewing it critically for important intellectual content; and final approval of the version to be published. **Sarita Bonagurio Gallo:** conception and design of the work, acquisition, financial, analysis, interpretation of data for the work. Drafting the work and reviewing it critically for important intellectual content; and final approval of the version to be published; and agreement to be accountable for all aspects of the work in ensuring that questions related to the accuracy or integrity of any part of the work are appropriately investigated and resolved.

## Funding

This work was supported by Fundação de Amparo à Pesquisa do Estado de São Paulo, 2017/20555‐8; Coordenação de Aperfeiçoamento de Pessoal de Nível Superior.

## Conflicts of Interest

The authors declare no conflicts of interest.

## Data Availability

The data that support the findings of this study are available on request from the corresponding author. The data are not publicly available due to privacy or ethical restrictions.
